# Outcomes in adult critically Ill cancer patients with and without neutropenia: a systematic review and meta-analysis of the Groupe de Recherche en Réanimation Respiratoire du patient d'Onco-Hématologie (GRRR-OH)

**DOI:** 10.18632/oncotarget.12165

**Published:** 2016-09-21

**Authors:** Marie Bouteloup, Sophie Perinel, Aurélie Bourmaud, Elie Azoulay, Djamel Mokart, Michael Darmon

**Affiliations:** ^1^ Medical-Surgical ICU, Hôpital Nord, Université Jean Monnet, Saint-Etienne, France; ^2^ Department of Public Health, Hygée Centre, Lucien Neuwirth Cancer Institut, Saint Priest en Jarez, France; ^3^ Medical ICU, Saint-Louis University Hospital, AP-HP, Paris, France; ^4^ Faculté de Médecine, Université Paris-Diderot, Sorbonne-Paris-Cité, Paris, France; ^5^ Anesthesiology and Intensive Care Unit, Institut Paoli Calmette, Marseille Cedex 9, France; ^6^ Thrombosis Research Group, EA 3065, Saint-Etienne University Hospital and Saint-Etienne Medical School, Saint-Etienne, France; ^7^ pour le Groupe de Recherche en Réanimation Respiratoire du patient d'Onco-Hématologie (GRRR-OH)

**Keywords:** prognosis, outcomes, hematologic, neoplasms, intensive care units

## Abstract

**PURPOSE:**

Whether neutropenia has an impact on the mortality of critically ill cancer patients remains controversial, yet it is widely used as an admission criterion and prognostic factor.

**METHODS:**

Systematic review and meta-analysis. Studies on adult cancer patients and intensive care units were searched on PubMed and Cochrane databases (2005-2015). Summary estimates of mortality risk differences were calculated using the random-effects model.

**RESULTS:**

Among the 1,528 citations identified, 38 studies reporting on 6,054 patients (2,097 neutropenic patients) were included. Median mortality across the studies was 54% [45–64], with unadjusted mortality in neutropenic and non-neutropenic critically ill patients of 60% [53–74] and 47% [41–68], respectively. Overall, neutropenia was associated with a 10% increased mortality risk (6%-14%; I^2^ = 50%). The admission period was not associated with how neutropenia affected mortality. Mortality significantly dropped throughout the study decade [−11% (−13.5 to −8.4)]. This mortality drop was observed in non-neutropenic patients [−12.1% (−15.2 to −9.0)] but not in neutropenic patients [−3.8% (−8.1 to +5.6)].

Sensitivity analyses disclosed no differences in underlying malignancy, mechanical ventilation use, or Granulocyte-colony stimulating factor use. Seven studies allowed the adjustment of severity results (1,350 patients). Although pooled risk difference estimates were similar to non-adjusted results, there was no significant impact of neutropenia on mortality (risk difference of mortality, 9%; 95% CI, −15 to +33)

**CONCLUSION:**

Although the unadjusted mortality of neutropenic patients was 11% higher, this effect disappeared when adjusted for severity. Therefore, when cancer patients become critically ill, neutropenia cannot be considered as a decision-making criterion.

## INTRODUCTION

The intensive care unit (ICU) admission of patients with cancer has long been controversial. Studies performed in the early 1990s demonstrated high mortality rates in cancer patients admitted to the ICU, especially among those with respiratory failure who required mechanical ventilation, those with neutropenic sepsis, and in hematopoietic stem cell transplant (HSCT) recipients [1, 2]. Over the past decade, however, studies reporting the prognosis and predictive factors for mortality in critically ill cancer patients have shown discrepant results [3–6]. Although mortality remains high when compared with the general population of critically ill patients, these studies have demonstrated meaningful ICU and hospital survival rates, as well as an improved survival over time for some of them [3–6]. Recent studies have also confirmed that the complete or partial remission of the underlying disease, lack of comorbidities, and good performance status are associated with an increased likelihood of survival, whereas some of the usual prognostic factors such as neutropenia failed to be associated with adverse outcomes [3, 4, 7].

Despite these results, the prognostic impact of neutropenia remains controversial. Hence, although meaningful survival has been described in neutropenic patients [6, 8], neutropenia remains a transient and expected immune dysfunction. Thus, neutropenia is a well-known factor of severe sepsis or acute respiratory failure [9] and is associated with a deterioration in respiratory status during neutropenia recovery [10]. Additionally, neutropenia is an independent risk factor of poor outcome in the general ICU population with severe sepsis or septic shock [11]. However, in cancer patients requiring ICU admission, the influence of neutropenia on outcome remains debatable. On one hand, several studies failed to demonstrate any impact of neutropenia on the outcome [3, 6], which might be explained by the frequent coexistence of several mechanisms of immune deficiency in these patients. On the other hand, previous studies in this field might have lacked the statistical power to detect any association between neutropenia and outcomes explaining the marginally significant influence of neutropenia in isolated reports [8]. Despite these uncertainties, neutropenia remains widely used as a prognostic factor.

The aim of this study was to assess the influence of neutropenia on the outcome of critically ill cancer patients. Secondary objectives included assessing the influence of neutropenia on the outcome of critically ill patients in pre-specified subgroups [according to the underlying tumor, period of admission, need for mechanical ventilation, and use of granulocyte colony-stimulating factor (G-CSF)].

## RESULTS

**Figure 1:**
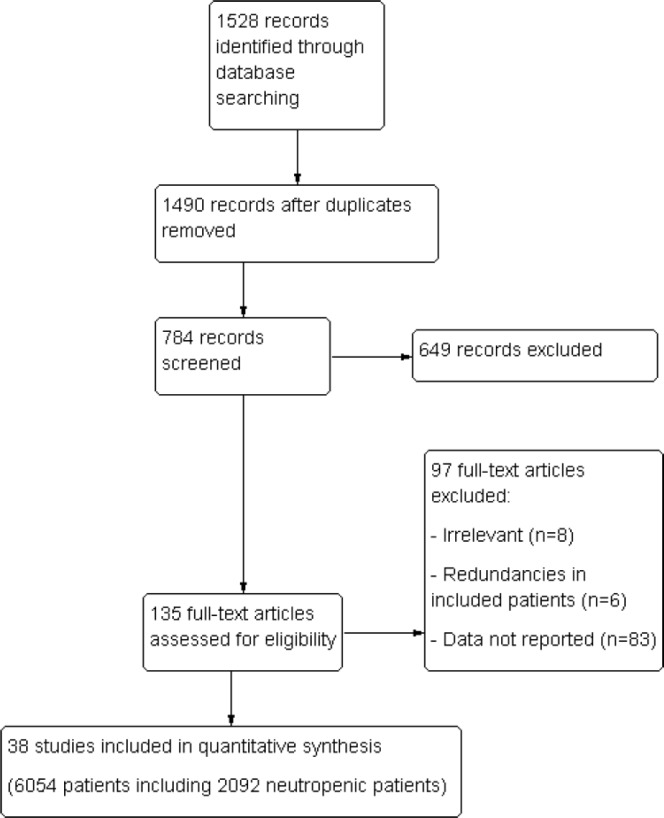
Flow chart of study selection

Our initial search yielded 1,528 citations, of which 38 were excluded for duplication. Among these records, 706 were excluded as irrelevant to the scope of this review. For the 784 remaining records, abstracts were carefully checked, and 135 full-text articles focusing on critically ill cancer patients' prognosis were scrutinized for further evaluation. Finally, 38 studies with a total of 6,054 patients (including 2,097 neutropenic patients and 2210 patients with solid tumors) fulfilled our eligibility criteria and were included (Figure 1) [6, 12–48]. Among included patients,

### Characteristics of included studies

Included studies were published from 2005 to 2015 with median inclusion period ranging from 1999 to 2010 (range, 1989–2013). Ten were prospective cohort studies, and five were multicentric cohort studies (Table S1). All studies were published in English (MeSH term of our research). Study populations varied across studies, including four studies focusing on cancer patients [21, 23, 34, 39], fourteen on patients with hematological malignancies, and the remaining on various mixes of these underlying diseases. Outcome variables included ICU mortality in 13 studies, hospital mortality in 24 studies, and 6-month mortality in a single study.

### Outcomes

Median mortality across all studies was 54% (45%–64%). Median mortality in neutropenic and non-neutropenic patients was of 60% (53%–74%) and 47% (41%–68%), respectively.

Overall mortality was 54.1% [3,275/6,054; 95% confidence interval (CI): 52.8%-55.4%]. The mortality of neutropenic and non-neutropenic patients was 62.8% (1,316/2,097; 95%CI: 60.7%–64.8%) and 49.5% (1,959/3,957; 95%CI: 47.9%–51.0%), respectively.

**Figure 2:**
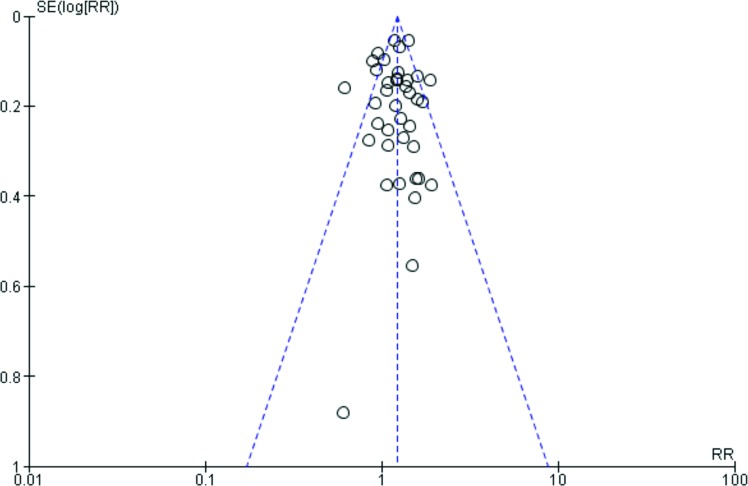
Funnel plot of included studies (SE: Standard error; RR: Relative risk)

**Figure 3:**
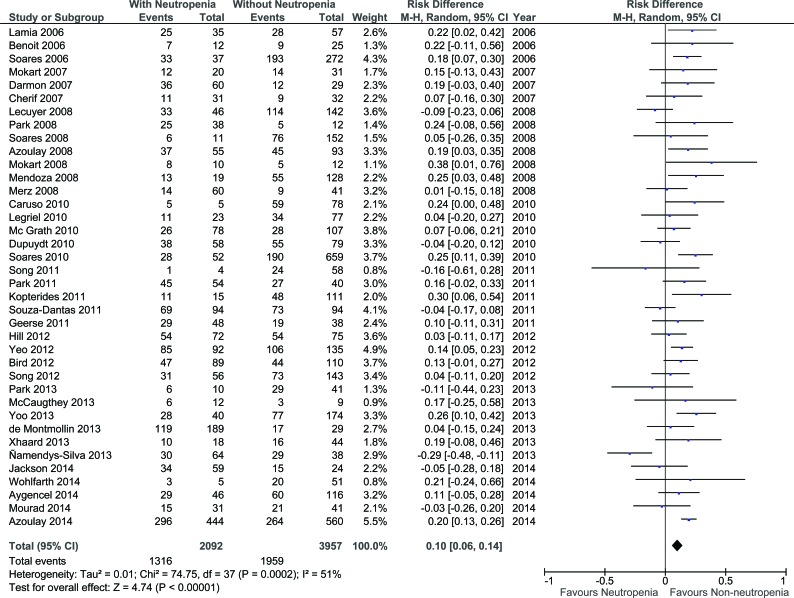
Summary of risk difference in included studies according to neutropenia (CI: confidence interval)

Funnel plot analysis failed to identify publication bias (Figure 2). Overall, neutropenia was associated with a 10% increased risk of mortality (95%CI 6%–14%; *P* - 0.0002; Figure 3).

### Influence of neutropenia in pre-defined subgroups

#### Influence of underlying malignancy

**Figure 4:**
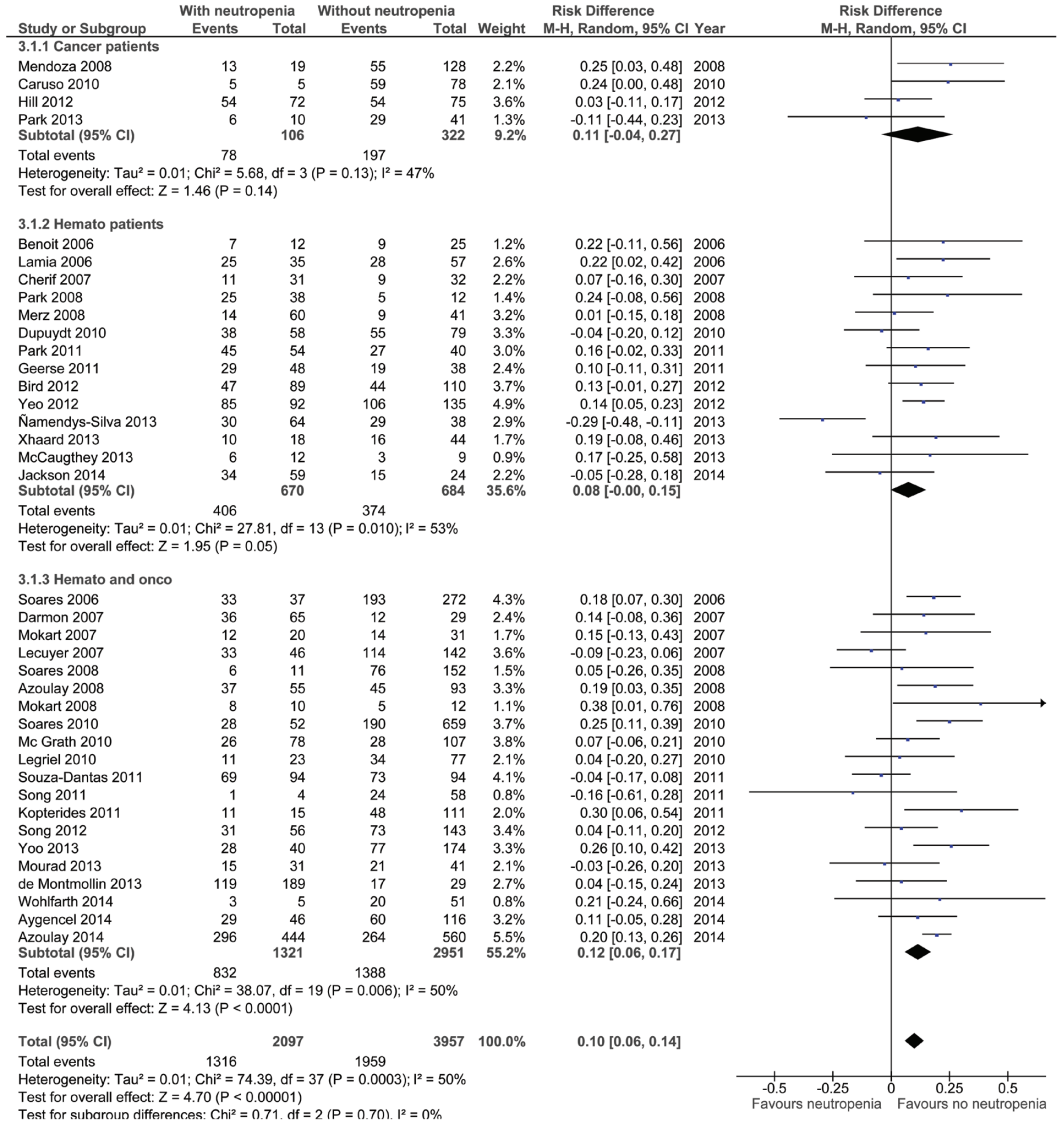
Summary of risk difference in included studies according to neutropenia and underlying malignancy (CI: confidence interval)

When analyzed separately, underlying malignancy did not modify the influence of neutropenia on the outcome (respective risk difference of mortality in neutropenic patients of 11% (95%CI: −4% – +27%), 8% (95%CI: 0% – +15%), and 12% (95%CI: 6% – 17%) in patients with solid tumors (*N* - 428), hematological malignancy (*N* - 1,354), or both (*N* - 4,272), respectively (Figure 4).

### Influence of ICU admission period

**Figure 5:**
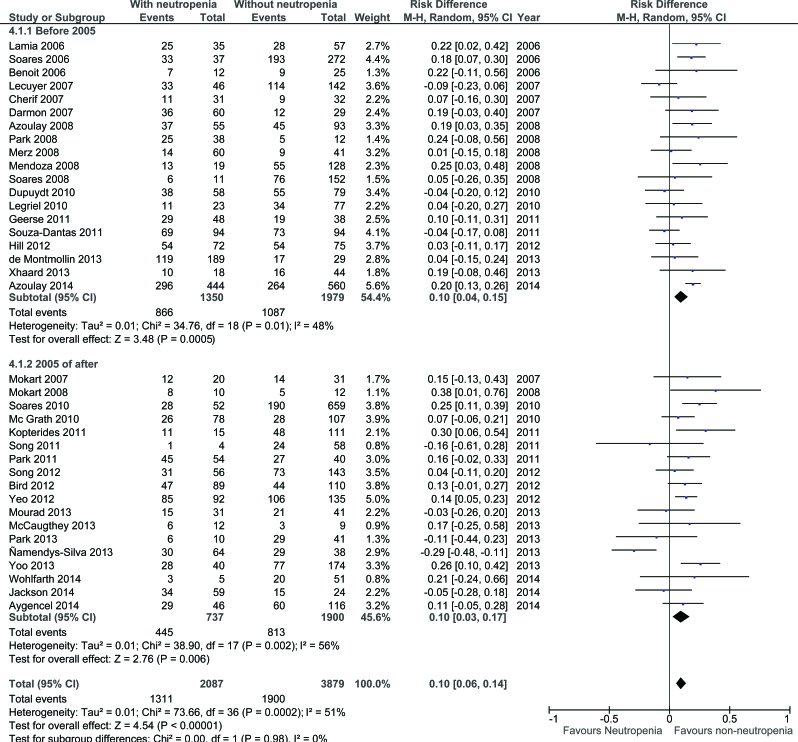
Summary of risk difference in included studies according to neutropenia and inclusion period (before and after 2005) A single study failing to report inclusion period was excluded for this analysis. CI: confidence interval

Mortality according to the inclusion period is displayed in Figure 5 and Figure S1. According to the inclusion period (one single study did not mention inclusion period and was excluded), the mortality in studies including patients before 2005 was 58.7% (95%CI: 57.0%–60.3%) compared with 47.8% after 2005 (95%CI: 45.8%–49.6%; difference: −11.0%; 95%CI: −13.5 to −8.4; *P* < 0.001). The mortality of neutropenic patients was 64.1% (95%CI: 61.6%–66.7%] before 2005 *vs.* 60.4% after 2005 (95%CI: 56.8%–63.9%; difference: −3.8%; 95%CI: −8.1 to +5.6; *P* - 0.09). The mortality of non-neutropenic patients was of 54.9% (95%CI: 52.7%–57.1%) before 2005 *vs.* 42.8% after 2005 (95%CI: 40.6%–45.0%; difference: −12.1%; 95%CI: −15.2% to −9.0%; *P* < 0.001).

The time of admission, therefore, did not modify the influence of neutropenia on the outcome (respective risk difference of mortality in neutropenic patients of 10% [95%CI: 4%-15%] before 2005 *vs.* 10% [95%CI: 3%-17%]).

### Influence of mechanical ventilation

In the included studies, the median rate of mechanical ventilation was 63% [52%-78%]. Influence of neutropenia was assessed according to tertiles of mechanical ventilation (< 56%, 56%–78%, and > 78%; Figure S2). Despite a high rate of patients included in the studies with a higher rate of mortality (2,458, including 1,022 neutropenic patients), the influence of neutropenia was no longer significantly associated with outcome in the third tertile (risk of mortality of 8%; 95%CI: −2% to +18%).

### Influence of G-CSF

Only four studies (349 patients, including 120 neutropenic patients) reported on the use of G-CSF. Use of G-CSF ranged from 8% to 100% of neutropenic patients, and evaluation of the influence of neutropenia in these studies was impaired by the lack of statistical power in this analysis (Figure S3).

### Adjusted influence of neutropenia

Overall, seven studies including 1,350 patients (642 patients with neutropenia) reported the adjusted influence of neutropenia on mortality. Although pooled risk difference estimates were similar to non-adjusted results, the overall influence of neutropenia was non-statistically significant (risk difference in mortality 9%; 95%CI: −15 to +33; *P* - 0.48; Figure S4).

### Factors associated with observed heterogeneity

Post-hoc meta-regression was performed in way to identify factors associated with observed heterogeneity. None of the recorded factors was significantly associated with changes in mortality associated with neutropenia (Table S2). Nevertheless, there was a non-significant trend toward a higher influence of neutropenia over mortality in studies with higher rate of solid tumor patients (Figure S5).

## DISCUSSION

This systematic review suggests neutropenia to be associated with a raw increase in mortality in critically ill patients. According to our results, the prognostic impact of neutropenia was unchanged when stratifying for malignancy, a period of admission, use of mechanical ventilation or G-CSF. Lastly, a significant improvement in overall prognosis was observed in the overall population of critically ill cancer patients and in non-neutropenic critically ill patients; meanwhile, the prognosis of neutropenic patients remained unchanged during the study period. Although the unadjusted mortality of patients with neutropenia was higher by 10%, this effect disappeared when results were adjusted for severity.

Neutropenia remains an accepted side effect of most treatments administered to cancer patients [49]. Despite being a transient and expected immune dysfunction, the duration of which is influenced by factors such as sepsis, lung injury, response to chemotherapy, underlying malignancy, and tumor progression or stage. Neutropenia is associated with complications that include severe sepsis [5], acute respiratory failure [50] and specific conditions such as neutropenic enterocolitis [51]. Although immune defects associated with neutropenia are likely to influence the outcome of critically ill patients, the results of studies in this field remain controversial. In the general ICU population, neutropenia remains associated with a poor outcome, especially in patients with severe sepsis [11]. In critically ill cancer patients, however, several recent studies failed to demonstrate any impact of neutropenia on the outcome [3, 6]. The numerous mechanisms of immune deficiency in these patients, along with the prognostic influence of disease severity or need for organ support therapies might explain these negative findings. Our results, however, suggest that neutropenia is associated with a 10% increase in overall mortality in this setting and that previous studies with negative findings may have been related to a lack of statistical power. On the other hand, the overall short-term prognosis reported in the analyzed studies remains meaningful (mortality, 60%; 95%CI: 53%–74%), and the influence of neutropenia was no longer significant after adjusting for confounders. The pooled point estimate of mortality (risk difference of mortality, +9%) along with the limited number of patients (1,350) and the wide confidence interval (−15% to +33%) suggests, however, a lack of statistical power and precludes any firm conclusion regarding this latter analysis. Despite this limitation, and that our results suggest neutropenia may be viewed as a risk factor of poor outcome in this specific population, it cannot justify the denial of ICU admissions for critically ill cancer patients.

Another interesting finding of our results is related to the changes in overall mortality according to the study period. Hence, a significant decrease in overall mortality was noted during the decade in the overall population (according to study inclusion period; *P* < 0.001) and in non-neutropenic patients according to a study inclusion period. Conversely, study inclusion period had little influence on critically ill neutropenic patients. Although, inclusion period in the included studies was too wide to allow for any definite conclusions, which may suggest that the survival gain suggested by previous studies [4, 6, 7, 52] may be limited in neutropenic patients. Additional studies are needed to confirm our results and to identify room for improvements in the management of this specific population.

This study has several important limitations. Firstly, despite the biological plausibility, this study at best demonstrated a statistical association between neutropenia and mortality. Secondly, the observed association is dependent upon observational studies and might have been affected by allocation bias not taken into account by our analysis. However, it must be noted that the point estimate of pooled adjusted analyzes was consistent with the unadjusted impact of neutropenia. Moreover, study inclusion period was estimated using median inclusion period. This surrogate is however imperfect; a few studies being performed over large period. Our results are however in line with previous studies suggesting progressive improvement of critically-ill cancer patients' prognosis over time [4, 6, 7, 52] and suggest limited improvement in neutropenic patients that may deserve to be confirmed by future studies in this field. Last, although no factor was identified as significantly associated with observed heterogeneity, meta-regression suggests higher rate of solid tumors to be associated with higher influence of neutropenia on outcome. The limited number of studies with high rate of solid tumors however limits statistical power of this analysis. Additional studies are therefore needed to confirm this finding.

In conclusion, this systematic review suggests a meaningful survival in neutropenic critically ill patients. Nevertheless, this study suggests a higher risk of death of 10% (6%–14%) in neutropenic critically ill cancer patients. Neither underlying malignancy, period of admission, use of mechanical ventilation, or use of G-CSF significantly influenced this result. Additional studies are needed to confirm our findings and to identify room for improvement in the management of these patients. Meanwhile, the meaningful survival of neutropenic patients in the reported studies strongly suggest that ICU admission denial based upon neutropenia should be discouraged.

## MATERIAS AND METHODS

This systematic review and meta-analysis were performed according to the guidelines of the Meta-analysis of Observational Studies in Epidemiology [53] and the PRISMA initiative (http://www.prisma-statement.org/). The study was registered in the PROSPERO database (CRD42015026347).

### Study outcome

The aim of this meta-analysis was to determine the prognostic impact of neutropenia on the outcome of critically ill cancer patients.

### Search strategy and eligibility assessment

First, public-domain databases PubMed and the Cochrane databases were searched using exploded medical subject headings and the appropriate corresponding keywords: “NEOPLASM” or “MALIGNANCY” or “CANCER” AND “INTENSIVE CARE UNIT” or “ICU”. The research was restricted to articles in English and studies involving humans and published from May 2005 to May 2015. Abstracts were carefully checked, and studies focusing on children or patients aged lower than 18 years, case reports, and studies failing to focus on critically ill patients were excluded.

All remaining references were then downloaded for consolidation, elimination of duplicates, and further analysis. Four authors (MB, SP, DM, and MD) independently evaluated the eligibility of all studies identified in the initial research. Lastly, studies with explicit redundancies were only included once.

### Data extraction and quality assessment

Four authors (MB, SP, DM, and MD) performed data extraction, working in pairs. Disagreements were resolved by discussion among authors and by adjudication of a third evaluator in case of persistent disagreement.

For each included trial, information was extracted on the following items: study design, study setting, follow-up period, study population, the proportion of HSTC recipients, the proportion of allogeneic HSCT recipients, the number of included patients, the number of patients with neutropenia, the outcome of patients with and without neutropenia.

ICU admission period was defined as the median inclusion year.

Risk of bias was assessed using the Cochrane's Tool to Assess Risk of Bias in Cohort Studies (http://methods.cochrane.org/bias/sites/methods.cochrane.org.bias/files/uploads/Tool%20to%20Assess%20Risk%20of%20Bias%20in%20Cohort%20Studies.pdf).

### Statistical analysis

Results were analyzed using Review Manager 5.1 (Cochrane Collaboration, Oxford, UK). Overall mortality of included patients and mortality in included studies are reported as medians (interquartile ranges). Publication bias was assessed by visually inspecting the funnel plot and summary estimates of risk differences were calculated using the random-effects model.

To enable data comparison, we transformed illness severity scores (SAPS II and APACHE III) into the equivalent APACHE II score, using a previously described methodology [54].

Four subgroup analyses were preplanned, and they included the influence of neutropenia according to underlying malignancy (solid tumor, hematological malignancy, or both), median ICU admission periods in the included studies, the use of mechanical ventilation, and the use of G-CSF. The last subgroup analysis on the duration of neutropenia could not be performed because this variable was unreported in most of the selected manuscripts.

A *P* value of less than 0.05 was considered statistically significant. Cochran's Chi^2^ test and I^2^ test for heterogeneity were used to assess inter-study heterogeneity [24]. The Chi^2^ test assesses whether observed differences among results are compatible with chance alone, and the I^2^ describes the percentage of the variability in effect estimates that results from heterogeneity rather than from sampling error. An I^2^ test for heterogeneity above 0.25 was considered to indicate moderate heterogeneity. Statistically significant heterogeneity was considered present at Chi^2^ *P* < 0.10 and I^2^ > 50%.

Last, since significant heterogeneity was observed, a post-hoc meta-regression was undertaken in way to identify factors that may be associated with the observed heterogeneity. This analysis was performed using R software (https://www.r-project.org/), Metafor package (https://cran.r-project.org/web/packages/metafor/metafor.pdf).

## SUPPLEMENTARY MATERIAL



## Abbreviations

ICU: Intensive care unit
Hematopoietic stem cell transplant: (HSCT)
Granulocyte-colony stimulating factor: (GCSF)
