# Supplementary material for: Outcomes in adult critically Ill cancer patients with and without neutropenia: a systematic review and meta-analysis of the Groupe de Recherche en Réanimation Respiratoire du patient d'Onco-Hématologie (GRRR-OH)
Source: Oncotarget. 2016 Sep 21;8(1):1860–70. doi: 10.18632/oncotarget.12165 (PMC5352103; doi:10.18632/oncotarget.12165)
Supplement: Supplementary file 1 [file oncotarget-08-1860-s001.pdf]

Outcomes in adult critically Ill cancer patients with and without neutropenia: a systematic review and meta-analysis

Supplementary Material

Table S1. Studies included in the meta-analysis and their characteristics.

Risk of bias was assessed using the Cochrane's Tool to Assess Risk of Bias in Cohort Studies. This tool evaluates eight components and result is reported as number of component considered at low risk of bias for each study.

Author, year	Follow up	n	APACHE II equivalent	Studied population	Allogeneic SCT (%)	Solid tumors (%)	Mortality (%)	Risk of bias
Lamia, 2006	Hospital	92	30	Hematological malignancies	11	0%	49	5
Benoit, 2006	Hospital	37	23	Hematological malignancies	0	0%	36	5
Soares, 2006	6 months	309	22	Both	0	76%	71	7
Darmon, 2007	Hospital	94	28	Both	0	7%	41	5
Cherif, 2007	ICU	63	20	Hematological malignancies	0	0%	28	6
Mokart, 2007	Hospital	51	26	Both	10	21%	45	5
Lecuyer, 2007	Hospital	188	NA	Both	0	30%	80	7
Park, 2008	ICU	50	26	Hematological malignancies	NA	0%	42	6
Soares, 2008	Hospital	163	25	Both	0	85%	50	7
Azoulay, 2008	Hospital	148	NA	Both	18	18%	48	6
Mokart, 2008	Hospital	22	24	Both	9	50%	42	5
Mendoza, 2008	Hospital	147	NA	Solid tumours	0	100%	43	5
Merz, 2008	ICU	101	28	Hematological malignancies	NA	0%	22	5
Caruso, 2010	Hospital	83	30	Solid tumours	0	100%	76	5
Legriel, 2010	Hospital	100	29	Both	14	26%	44	5
Mc Grath, 2010	ICU	185	19	Both	NA	38%	26	5
Dupuydt, 2010	Hospital	137	26	Hematological malignancies	15	0%	70	7
Soares, 2010	Hospital	711	21	Both	0,3	93%	29	7
Park, 2011	ICU	94	32	Hematological malignancies	13	0%	67	4
Song, 2011	ICU	62	28	Both	NA	21%	41	5
Kopterides, 2011	ICU	126	18	Both	NA	71%	38	5
Geerse, 2011	ICU	86	29	Hematological malignancies	23	0%	50	5

Souza-Dantas, 2011	Hospital	188	31	Both	0	32%	78	7
Bird, 2012	6 months	199	21	Hematological malignancies	NA	0%	40	5
Song, 2012	Hospital	199	NA	Both	11	52%	51	6
Hill, 2012	Hospital	147	26	Hematological malignancies	5	0%	72	5
Yeo, 2012	ICU	227	19	Hematological malignancies	5	0%	78	7
Mc Caugthey, 2013	ICU	21	23	Hematological malignancies	10	0%	33	5
Yoo, 2013	Hospital	214	NA	Both	8	46%	44	7
de Montmollin, 2013	Hospital	218	NA	Both	8,3	16%	59	6
Park, 2013	ICU	51	17	Solid tumours	0	100%	70	5
Xhaard, 2013	ICU	62	NA	Hematological malignancies	0	0%	36	5
Namendys-Silva, 2013	Hospital	102	17	Hematological malignancy	4,9	0%	76	6
Mourad, 2014	ICU	72	29	Both	18	36%	51	5
Azoulay, 2014	Hospital	1004	9	Both	12	15%	47	5
Jackson, 2014	Hospital	83	21	Hematological malignancies	0	0%	62	4
Wohlfarth, 2014	ICU	56	27	Both	0	12%	39	5
Aygencel, 2014	ICU	162	23	Both	10	64%	52	6

Table S2. Factors associated with influence of neutropenia on outcome according to Meta-regression.

	β	se	I^2	Heterogeneity	p-value
Prospective study (vs. retrospective)	0.042	0.052	51.76%	0.0003	0.42
Multicenter study (vs. monocenter)	0.06	0.05	47.79	0.002	0.24
Medico-surgical population (vs. medical)	-0.04	0.05	51.71%	0.0003	0.34
Stem-Cell transplant recipient (%)	0.02	0.16	32.72%	0.09	0.89
Allogeneic SCT recipients (%)	-0.048	0.41	57.68%	0.0004	0.91
APACHE 2 Score of included patients	-0.003	0.004	47.72	0.007	0.52
Solid tumors (%)	0.1	0.07	48.42%	0.0005	0.12
Median inclusion period	-0.0035	0.007	52.21%	0.0003	0.62
Mechanical ventilation (%)	-0.09	0.11	50.36%	0.0002	0.39

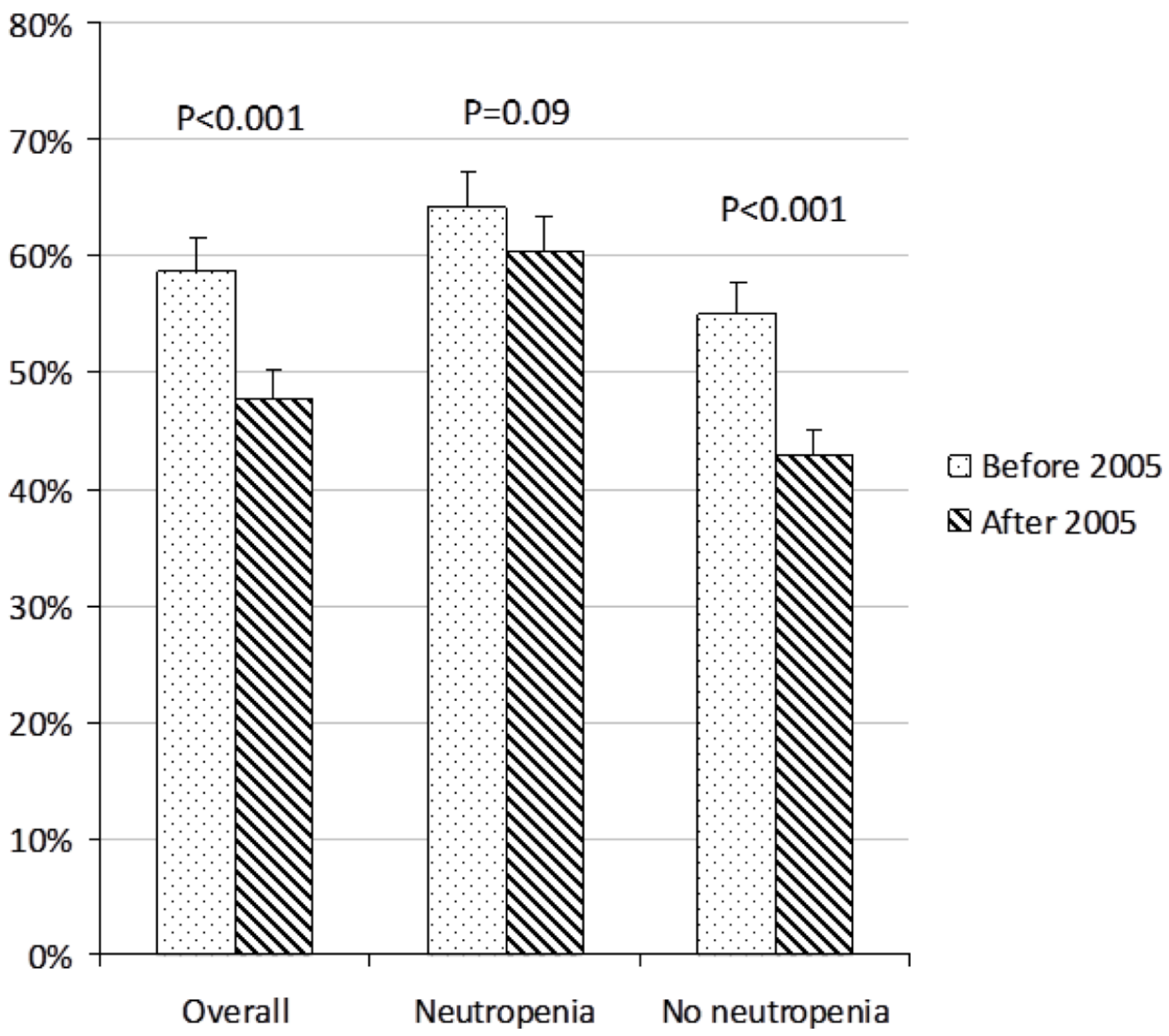

Figure S1. Mortality according to study inclusion in the overall population and in patients with and without neutropenia.

Figure S2

Study or Subgroup	With Neutropenia Events	With Neutropenia Total	Without Neutropenia Events	Without Neutropenia Total	Weight	Risk Difference M-H, Random, 95% CI
**6.1.1 MV<56%**						
Caruso 2010	5	5	59	78	2.1%	0.24 [0.00, 0.48]
Cherif 2007	11	31	9	32	2.2%	0.07 [-0.16, 0.30]
Geerse 2011	29	48	19	38	2.4%	0.10 [-0.11, 0.31]
Hill 2012	54	72	54	75	3.6%	0.03 [-0.11, 0.17]
Mc Grath 2010	26	78	28	107	3.8%	0.07 [-0.06, 0.21]
Merz 2008	14	60	9	41	3.2%	0.01 [-0.15, 0.18]
Mourad 2014	15	31	21	41	2.1%	-0.03 [-0.26, 0.20]
Soares 2010	28	52	190	659	3.7%	0.25 [0.11, 0.39]
Song 2012	31	56	73	143	3.4%	0.04 [-0.11, 0.20]
Xhaard 2013	10	18	16	44	1.8%	0.19 [-0.08, 0.46]
Yeo 2012	85	92	106	135	4.9%	0.14 [0.05, 0.23]
Yoo 2013	28	40	77	174	3.3%	0.26 [0.10, 0.42]
**Subtotal (95% CI)**		**583**		**1567**	**36.5%**	**0.12 [0.06, 0.17]**
Total events	336		661			
Heterogeneity: Tau² = 0.00; Chi² = 13.94, df = 11 (P = 0.24); I² = 21%						
Test for overall effect: Z = 4.33 (P < 0.0001)						
**6.1.2 56%<MV<78%**						
Aygencel 2014	29	46	60	116	3.2%	0.11 [-0.05, 0.28]
Benoit 2006	7	12	9	25	1.3%	0.22 [-0.11, 0.56]
Bird 2012	47	89	44	110	3.7%	0.13 [-0.01, 0.27]
Darmon 2007	36	60	12	29	2.3%	0.19 [-0.03, 0.40]
Dupuydt 2010	38	58	55	79	3.3%	-0.04 [-0.20, 0.12]
Jackson 2014	34	59	15	24	2.2%	-0.05 [-0.28, 0.18]
Lamia 2006	25	35	28	57	2.6%	0.22 [0.02, 0.42]
Legriel 2010	11	23	34	77	2.2%	0.04 [-0.20, 0.27]
McCaugthey 2013	6	12	3	9	0.9%	0.17 [-0.25, 0.58]
Mendoza 2008	13	19	55	128	2.2%	0.25 [0.03, 0.48]
Park 2011	45	54	27	40	3.0%	0.16 [-0.02, 0.33]
Soares 2008	6	11	76	152	1.5%	0.05 [-0.26, 0.35]
Song 2011	1	4	24	58	0.8%	-0.16 [-0.61, 0.28]
Wohlfarth 2014	3	5	20	51	0.8%	0.21 [-0.24, 0.66]
**Subtotal (95% CI)**		**487**		**955**	**29.8%**	**0.11 [0.05, 0.17]**
Total events	301		462			
Heterogeneity: Tau² = 0.00; Chi² = 11.64, df = 13 (P = 0.56); I² = 0%						
Test for overall effect: Z = 3.65 (P = 0.0003)						
**6.1.3 78%<MV**						
Azoulay 2008	37	55	45	93	3.3%	0.19 [0.03, 0.35]
Azoulay 2014	296	444	264	560	5.5%	0.20 [0.13, 0.26]
de Montmollin 2013	119	189	17	29	2.7%	0.04 [-0.15, 0.24]
Kopterides 2011	11	15	48	111	2.0%	0.30 [0.06, 0.54]
Lecuyer 2008	33	46	114	142	3.6%	-0.09 [-0.23, 0.06]
Mokart 2007	12	20	14	31	1.7%	0.15 [-0.13, 0.43]
Mokart 2008	8	10	5	12	1.1%	0.38 [0.01, 0.76]
Park 2008	25	38	5	12	1.4%	0.24 [-0.08, 0.56]
Park 2013	6	10	29	41	1.3%	-0.11 [-0.44, 0.23]
Soares 2006	33	37	193	272	4.3%	0.18 [0.07, 0.30]
Souza-Dantas 2011	69	94	73	94	4.1%	-0.04 [-0.17, 0.08]
Ñamendys-Silva 2013	30	64	29	38	2.9%	-0.29 [-0.48, -0.11]
**Subtotal (95% CI)**		**1022**		**1435**	**33.7%**	**0.08 [-0.02, 0.18]**
Total events	679		836			
Heterogeneity: Tau² = 0.02; Chi² = 49.14, df = 11 (P < 0.00001); I² = 78%						
Test for overall effect: Z = 1.61 (P = 0.11)						
**Total (95% CI)**		**2092**		**3957**	**100.0%**	**0.10 [0.06, 0.14]**
Total events	1316		1959			
Heterogeneity: Tau² = 0.01; Chi² = 74.75, df = 37 (P = 0.0002); I² = 51%						
Test for overall effect: Z = 4.74 (P < 0.00001)						
Test for subgroup differences: Chi² = 0.34, df = 2 (P = 0.84), I² = 0%						

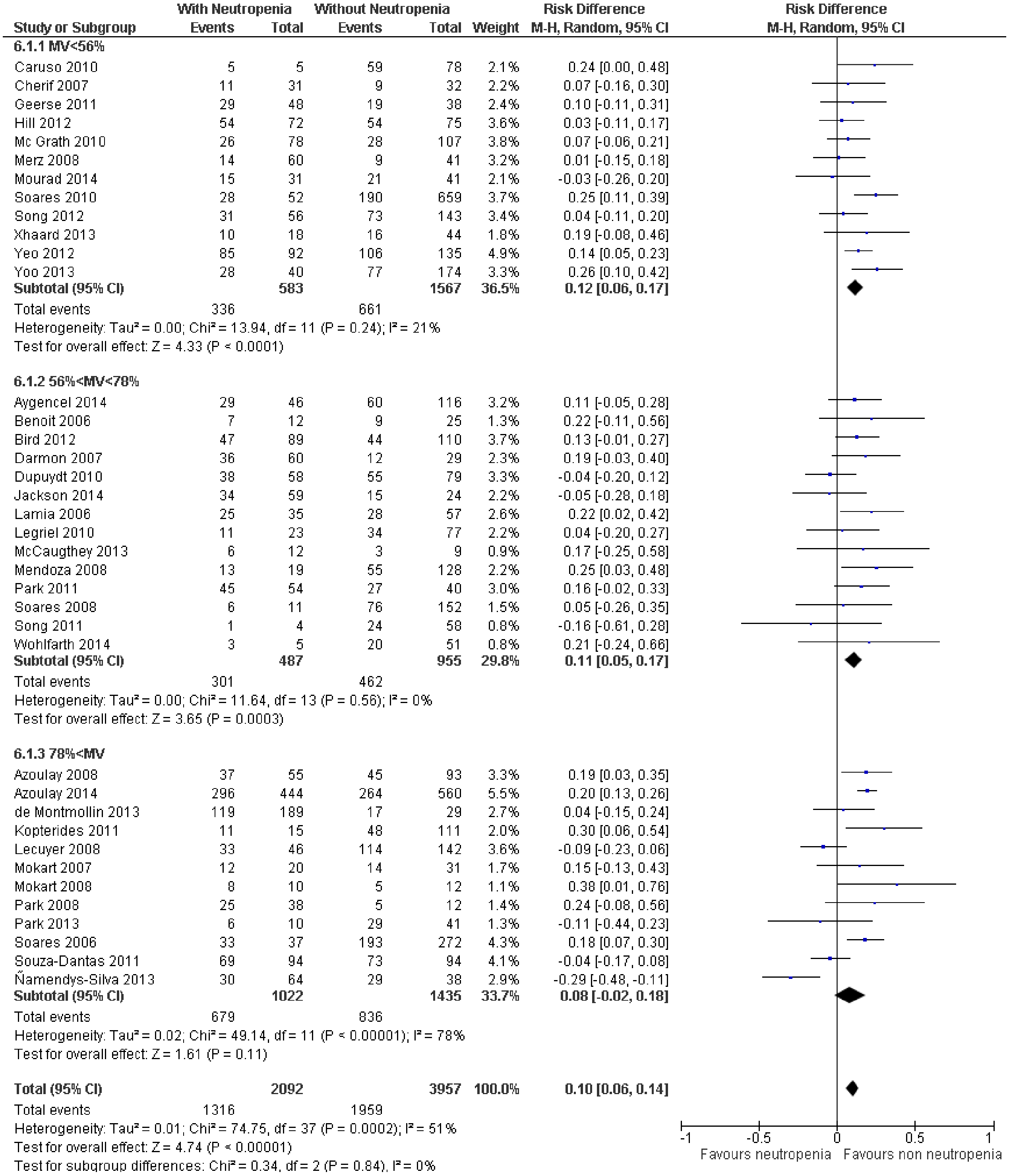

Figure S2. Summary of risk difference in included studies according to rate of mechanical ventilation. Studies were separated according to tertiles of mechanical ventilation requirement (<56%, 56-78%, >78%).

Figure S3.

Study or Subgroup	Neutropenia Events	Neutropenia Total	No neutropenia Events	No neutropenia Total	Weight	Risk Difference M-H, Random, 95% CI	Year
**7.1.1 G-CSF <50%**							
Lecuyer 2008	33	46	114	142	39.3%	-0.09 [-0.23, 0.06]	2008
Wohlfarth 2014	3	5	20	51	13.6%	0.21 [-0.24, 0.66]	2014
**Subtotal (95% CI)**		**51**		**193**	**52.9%**	**-0.02 [-0.26, 0.22]**	
Total events	36		134				
Heterogeneity: Tau² = 0.01; Chi² = 1.49, df = 1 (P = 0.22); I² = 33%							
Test for overall effect: Z = 0.15 (P = 0.88)							
**7.1.2 G-CSF >50%**							
Mokart 2008	8	10	5	12	17.6%	0.38 [0.01, 0.76]	2008
Jackson 2014	34	59	15	24	29.4%	-0.05 [-0.28, 0.18]	2014
**Subtotal (95% CI)**		**69**		**36**	**47.1%**	**0.14 [-0.28, 0.56]**	
Total events	42		20				
Heterogeneity: Tau² = 0.07; Chi² = 3.74, df = 1 (P = 0.05); I² = 73%							
Test for overall effect: Z = 0.66 (P = 0.51)							
**Total (95% CI)**		**120**		**229**	**100.0%**	**0.05 [-0.15, 0.24]**	
Total events	78		154				
Heterogeneity: Tau² = 0.02; Chi² = 6.30, df = 3 (P = 0.10); I² = 52%							
Test for overall effect: Z = 0.48 (P = 0.63)							
Test for subgroup differences: Chi² = 0.42, df = 1 (P = 0.52), I² = 0%							

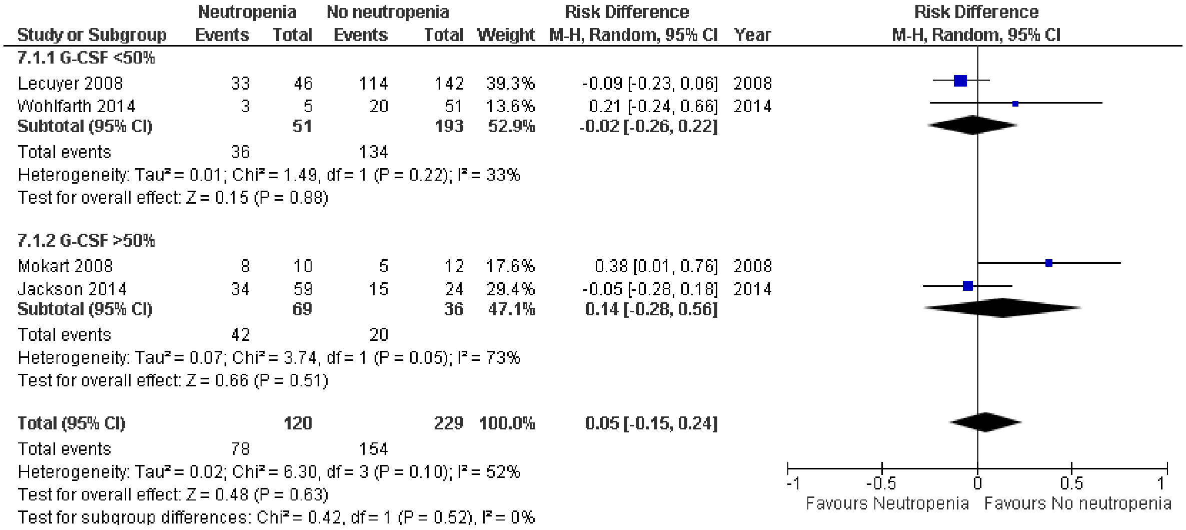

Figure S3. Summary of risk difference in included studies according to use of G-CSF.

Figure S4.

Study or Subgroup	Risk Difference	SE	Weight	Risk Difference IV, Random, 95% CI	Year
Vandijk 2008	0.0862	0.3679	11.2%	0.09 [-0.63, 0.81]	2008
Souza-Dantas 2011	0.2307	0.3403	13.1%	0.23 [-0.44, 0.90]	2011
Park 2011	-1.8326	0.9928	1.5%	-1.83 [-3.78, 0.11]	2011
Yeo 2012	0.1222	0.6128	4.0%	0.12 [-1.08, 1.32]	2012
Park 2013	-0.3369	1.1651	1.1%	-0.34 [-2.62, 1.95]	2013
Ñamendys-Silva 2013	-0.0564	0.2224	30.7%	-0.06 [-0.49, 0.38]	2013
Mokart 2015	0.239	0.1989	38.3%	0.24 [-0.15, 0.63]	2015
**Total (95% CI)**			**100.0%**	**0.09 [-0.15, 0.33]**	

Heterogeneity: $Tau^2 = 0.00$; $Chi^2 = 5.05$, df = 6 (P = 0.54); $I^2 = 0\%$
Test for overall effect: Z = 0.71 (P = 0.48)

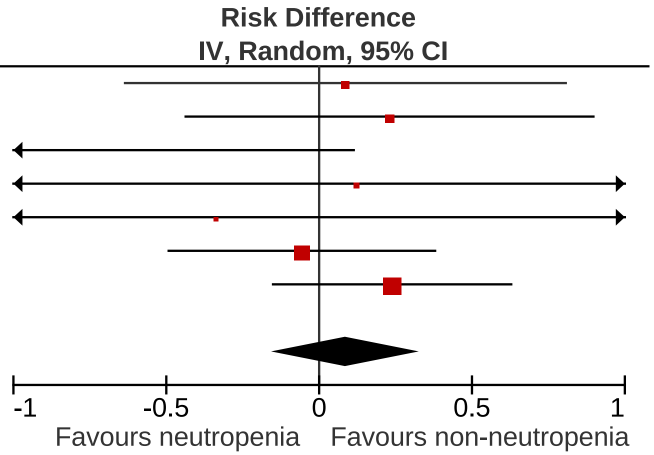

Figure S4. Adjusted influence of neutropenia on outcome.

Figure S5.

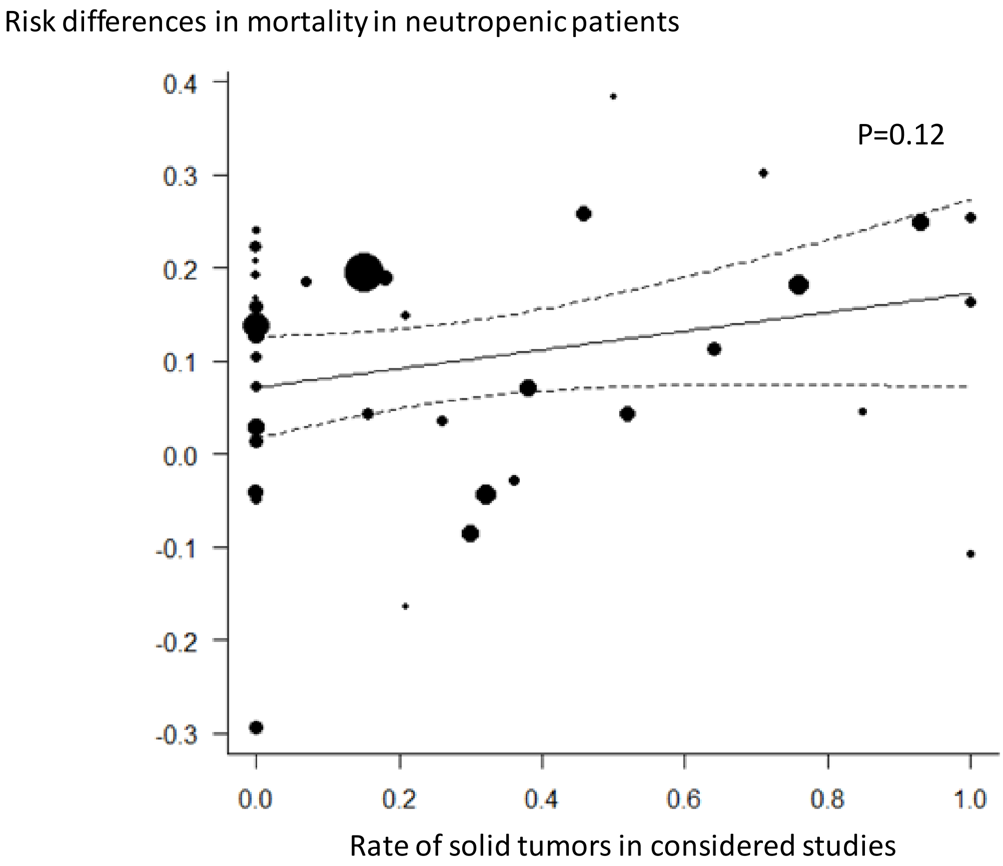

Figure S5. Influence of neutropenia on outcome according to rate of patients with solid tumors in considered studies.
